# Combined Short and Long-Read Sequencing Reveals a Complex Transcriptomic Architecture of African Swine Fever Virus

**DOI:** 10.3390/v13040579

**Published:** 2021-03-30

**Authors:** Gábor Torma, Dóra Tombácz, Zsolt Csabai, Norbert Moldován, István Mészáros, Zoltán Zádori, Zsolt Boldogkői

**Affiliations:** 1 Department of Medical Biology, Faculty of Medicine, University of Szeged, Somogyi B. u. 4., 6720 Szeged, Hungary; torma.gabor@med.u-szeged.hu (G.T.); tombacz.dora@med.u-szeged.hu (D.T.); csabai.zsolt@med.u-szeged.hu (Z.C.); moldovan.norbert@med.u-szeged.hu (N.M.); 2 Institute for Veterinary Medical Research, Centre for Agricultural Research, Hungária krt. 21, H-1143 Budapest, Hungary; meszaros.istvan@agrar.mta.hu (I.M.); zadori.zoltan@agrar.mta.hu (Z.Z.)

**Keywords:** African swine fever virus, ASFV, transcriptome, long-read sequencing, direct RNA sequencing

## Abstract

African swine fever virus (ASFV) is a large DNA virus belonging to the Asfarviridae family. Despite its agricultural importance, little is known about the fundamental molecular mechanisms of this pathogen. Short-read sequencing (SRS) can produce a huge amount of high-precision sequencing reads for transcriptomic profiling, but it is inefficient for comprehensively annotating transcriptomes. Long-read sequencing (LRS) can overcome some of SRS’s limitations, but it also has drawbacks, such as low-coverage and high error rate. The limitations of the two approaches can be surmounted by the combined use of these techniques. In this study, we used Illumina SRS and Oxford Nanopore Technologies LRS platforms with multiple library preparation methods (amplified and direct cDNA sequencings and native RNA sequencing) for constructing the ASFV transcriptomic atlas. This work identified many novel transcripts and transcript isoforms and annotated the precise termini of previously described RNAs. This study identified a novel species of ASFV transcripts, the replication origin-associated RNAs. Additionally, we discovered several nested genes embedded into larger canonical genes. In contrast to the current view that the ASFV transcripts are monocistronic, we detected a significant extent of polycistronism, although a large proportion of these transcripts are expressed in low abundance. A multifaceted meshwork of transcriptional overlaps was also discovered.

## 1. Introduction

High-throughput massively parallel sequencing has revolutionized modern biology by facilitating the explosive growth of genomics and transcriptomics. Next-generation short-read sequencing (SRS) has allowed producing a tremendous volume of data with unprecedented speed. Currently, Illumina rules the SRS market. RNA studies are hampered by the limitations of SRS that cannot sufficiently cope with the complexity of large transcriptomes [1]. Third-generation long-read sequencing (LRS) can overcome many of the drawbacks of SRS, which include the inefficiency to make a distinction between transcription isoforms, the low-efficiency of detecting long RNA molecules, and transcriptional overlaps [2]. The LRS platforms have two main disadvantages: relatively low throughput and high error rate. However, with the combined use of these technologies, high-quality and high-throughput data can be produced on full-length transcripts [3].

African swine fever (ASF) is a highly lethal hemorrhagic disease of domestic pigs. Its causative agent is the ASF virus (ASFV) that is the sole member of the Asfarviridae family [4]. The natural hosts of the virus are warthogs, bush pigs and soft ticks of the genus *Ornithodoros*, which makes ASFV the only DNA virus that can infect both mammals and arthropods [5]. To date, 24 genotypes of the virus have been identified [6]. Genotypes I and II have caused severe worldwide pandemics. Since effective vaccine or antiviral drugs are unavailable against the virus at this moment, it unquestionably presents the largest worldwide economic threat to the animal industry in recorded history.

ASFV belongs to nucleocytoplasmic large DNA viruses. Even though some form of nuclear replication is detected in the early phase of the infection [7], it mainly replicates itself in the cytoplasm. The genetic organization of ASFV best resembles that of the poxviruses [8,9]. The viral genome is ~170–190 kbp long and contains covalently closed terminal repeats at its ends. The majority of genome variations are localized among the genes belonging to the multigene families (MGFs) at the genome termini [8]. Genetic analyses have revealed over 160 open reading frames (ORFs) in the viral genome, although the exact number of protein-coding genes is yet unknown.

With a few exceptions [10], most of the ASFV ORFs have been reported to be transcribed as monocistronic RNA molecules [9,10,11,12], which if so, represent a different principle of transcriptome organization than those of other large DNA viruses, which express a large number of multigenic transcripts (e.g., poxviruses, herpesviruses, and baculoviruses; [11,13,14,15,16,17]. ASFV ORFs are compactly laid in both strands with very short intergenic regions or with minimal overlaps with tail-to-head (→→), head-to-head (→←) or tail-to-tail (←→) arrangements [8,18]. The regulatory elements and promoters of genes usually overlap with the transcribed regions of the neighboring genes. Therefore, the transcriptional activity of genes may interfere with each other. Very little information is available on the functioning of ASFV promoters. At the late phase of infection, p30 and DNA polymerase promoter have higher activity than that of the p72 promoter regulating the transcription of the most abundant viral protein p72 [19]. The four-nucleotide core TATA sequence appears to be an essential motif in most late promoters [20].

Molecular biological analysis of ASFV is not easy because most wild-type viral strains cannot be propagated in established cell lines [21]. The virus replicates relatively well in porcine primary alveolar macrophages (PAMs) in vitro, although the sensitivity of naïve PAM culture to ASFV infection varies batch by batch [22].

The transcription kinetics of ASFV has only been studied in detail in a few cases. The temporal regulation of ASFV gene expression appears to be similar to the transcription cascade described in poxviruses [9,23], in which four classes of the consecutively transcribed RNAs have been described. The ASFV immediate-early (IE) and early (E) genes are mainly synthesized before the DNA replication, whereas intermediate (I) and late (L) genes are transcribed after the onset of DNA replication [9]. Transcripts are initiated and terminated at precise sites upstream and downstream of the ORFs, respectively [24]. The transcribed RNAs have 5′-cap structures, and 3′-poly(A) tails with an average length of 33 nucleotides added by the viral capping enzyme complex and the poly(A) polymerase, respectively [25]. Jaing and coworkers have carried out a survey on the entire ASFV transcriptome using SRS-based RNA-seq [26]. Approximately 60% of the ORFs (109 genes) were detected from the circulating monocytes of highly pathogenic Georgia 2007/1 infected pigs. Although this study has provided valuable information about highly expressed ASFV genes, it has not supplied sufficient data to construct a detailed transcriptional map of the virus. In a recent study, Cackett and coworkers reported an SRS analysis of the transcriptome of a non-host cell (Vero)-adapted genotype I ASFV strain (BA71V) containing large deletions at both ends of the viral genome [12].

The main objective of this study is to gather additional information about the transcriptomic architecture of a wild-type ASFV in natural host cells by applying a dual sequencing multi-technique approach. We cataloged all types of the transcript, including mRNAs, non-coding RNAs (ncRNAs) and transcript isoforms, and carried out a semiquantitative analysis of the expression levels of the transcripts at different stages of viral infection. Clarifying the functional elements of the viral genome is vital for a better understanding of the unique biology of this dangerous pathogen.

## 2. Materials and Methods

**Figure 1:**
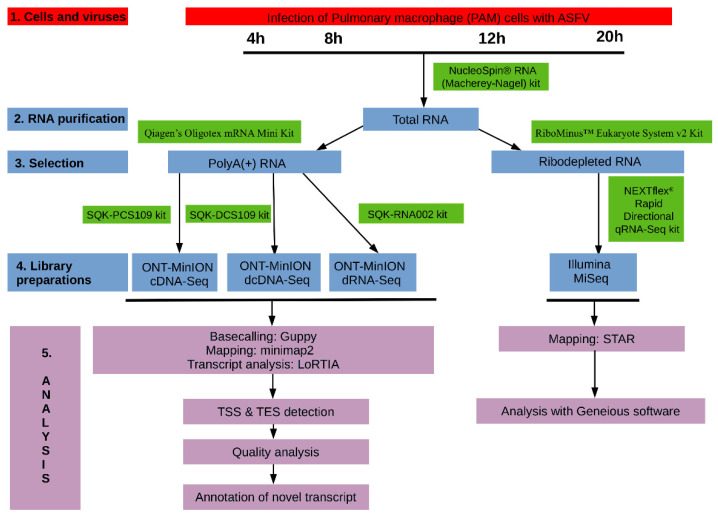
Wetlab and bioinformatics workflow used for the analysis of African swine fever virus (ASFV) transcriptome.

The experimental design utilized in this study is visualized in Figure 1.

### 2.1. Cells, Viruses and Infection

Porcine alveolar macrophage (PAM) cells were freshly harvested from swine lungs according to the OIE Manual’s instructions [27]. PAM cells were used for the propagation of the highly virulent ASFV_HU_2018 isolate of the African swine fever virus (Accession Number: MN715134.1). PAM cells were grown in RPMI 1640-containing L-glutamine (Lonza, Basel, Switzerland) medium supplemented with 10% fetal bovine serum (Euro Clone, Pero, Italy), 1% Na-pyruvate (Lonza), 1% non-essential amino acid solution (Lonza), and 1% antibiotic-antimycotic solution (Thermo Fisher Scientific, Waltham, MA, USA) at 37 °C in 5% CO_2_ in air gas phase. The infectious titer of serially diluted viral stock was calculated using an immunofluorescence (IF) assay as described earlier [22]. PAMs were cultivated in 6-well plates at a density of 3.3 × 10^5^ cells and infected at a multiplicity of infection (MOI) of 10 plaque-forming units per cell at 4 h after cell seeding. The supernatant was replaced with a fresh medium after 1 h post-infection (hpi). Infected PAM cells were harvested at 4, 8, 12, and 20 hpi, whereas mock-infected cells were harvested at 20 hpi IF assay was used for monitoring the efficiency of infection in an infected control well fixed at 20 hpi.

### 2.2. Infection Efficiency

The length of the ASFV infection cycle length is approximately 18–22 h [19,28], yet virion production very often peaks around 72 h post-infection in PAM cells [21,29,30] since only a relatively low percentage of naïve PAM cells can be initially infected [31]. Since our intention was to characterize the dynamics of transcription, we harvested “first round” infected cells of two animals at 4, 8, 12 and 20 hpi. This sampling scheme allowed us to exclude a “second round” of infection in originally non-infected cells, which would lead to false conclusions about the kinetics of the viral transcripts. Indeed, infection efficiency remained at approximately 20% at 20 hpi despite the high viral titer (MOI = 10) applied for the infection.

### 2.3. RNA Purification

#### 2.3.1. Extraction of Total RNA

We used the NucleoSpin^®^ RNA (Macherey–Nagel, Düren, Germany) kit for isolation of the total RNA from the samples as was described in our previous publications [32]. Briefly, samples were incubated with a lysis buffer (supplied by the kit), then DNase I treatment was carried out. Purified RNA samples were eluted from the silica membrane in nuclease-free water. Samples were stored at −80 °C until further use. The total RNAs were used directly for the “amplified cDNA protocol” from ONT.

#### 2.3.2. Purification of Polyadenylated RNAs

For the direct RNA (dRNA) and direct cDNA (dcDNA) sequencing approaches, the poly(A)+ fraction of the total RNAs were extracted. This process was carried out with the Qiagen’s Oligotex mRNA mini kit using spin columns according to the kit’s manual.

#### 2.3.3. Removal of the Ribosomal RNAs

The RiboMinus™ Eukaryote System v2 (Thermo Fisher Scientific) was used to obtain rRNA-free RNA samples, which is required by the applied Illumina library preparation approach. For explanation, the NEXTFLEX^®^ rapid directional RNA-Seq Kit 2.0 was used for the preparation of strand-specific single-end or paired-end RNA libraries as recommended by the manufacturer. The following steps were used: RNA fragmentation, first-strand synthesis, second-strand synthesis, adenylation, adapter ligation, and PCR amplification. The ribodepletion was carried out according to the kit’s instructions.

### 2.4. Library Preparations

#### 2.4.1. Direct RNA Sequencing on MinION SpotON Flow Cells

Native RNA sequencing was carried out using the ONT SQK-RNA002 kit as described earlier [33,34]. In brief, polyA (+) RNA mixture (including 4, 8, 12, and 20 hpi) was reverse transcribed using oligo(dT) primer (from the ONT kit), NEBNext quick ligation reaction buffer and T4 DNA ligase, dNTPs (both from New England Biolabs, Ipswich, MA, USA), and enzyme and buffer from the Invitrogen’s SuperScript III kit. RNA-cDNA hybrids were washed. For this, Agencourt RNAClean XP beads (Beckman Coulter, Brea, CA, USA) were added to the samples (1.8 bead ratio (BR)), and they were mixed. The mixtures were incubated at room temperature on a HulaMixer™ (Thermo Scientific) for 5 min; then, they were placed in a magnetic separator (MagJET separation rack/Thermo Scientific) until the liquid cleared. The supernatant was discarded, and the nucleic acid-bound beads were washed with 70% ethanol two times. Finally, the samples were eluted in UltraPure™ DNase/RNase-free distilled water (Invitrogen, Carlsbad, CA, USA).

#### 2.4.2. Direct cDNA Sequencing on MinION Flow Cells

The same polyA (+) RNA mixture was used for the preparation of the dcDNA library as for dRNA sequencing. The library was generated with the ONT’s direct cDNA sequencing kit (SQK-DCS109) according to ONT’s protocol. Briefly, the reverse transcription (RT) reaction was conducted in the presence of anchored oligo(dT) primers (VN primer from the ONT Kit), dNTPs, buffer and enzyme from the Maxima H minus reverse transcriptase kit (Thermo Fisher Scientific), as well as strand switching primer (SSP, including in the ONT kit). To avoid RNA degradation, RNaseOUT™ (Thermo Fisher Scientific) was also added to the reaction. After cDNA synthesis, the RNA strands were digested using an RNase cocktail enzyme mix (Thermo Fisher Scientific). The sample was cleaned using the XP bead purification method with AMPure XP beads (0.8 BR) (Beckman Coulter). Samples were incubated for 5 min on the HulaMixer; then, they were cleaned as was described in the dRNA-seq section. The second cDNA strand was generated using LongAmp Taq master mix from New England Biolabs and the PNT’s PR2 primer. Double-stranded cDNAs were washed with XP beads then were handled with NEBNext Ultra II end-prep enzyme mix (New England Biolabs). After an XP bead-washing, the sample is ligated to the ONT adapter mix using blunt/TA ligation master mix (New England Biolabs). Finally, 200 fmol from the XP bead-purified sample was run on two SpotON Flow Cells.

#### 2.4.3. Amplified cDNA-Sequencing Using MinION Device

Total RNA samples from each time point (4, 8, 12, and 20 hpi) were sequenced individually using the ONT MinION device and the cDNA-PCR Barcoding protocol (SQK-PCS109 and SQK-PBK004). RT was carried out as described in the dcDNA protocol (above). The samples were amplified using the LongAmp Taq master mix. Low-input barcode primers (ONT’s SQK-PBK004 kit) were added to the samples with the aim of multiplexing the samples on the Flow Cells. After the PCR reaction, cDNAs were treated with an exonuclease, and they were finally cleaned with AMPure XP beads, as it was explained in the dcDNA paragraph.

#### 2.4.4. Amplified cDNA Sequencing Using Illumina MiSeq Sequencer

The viral transcriptome was also sequenced by using the Illumina SRS approach. The NEXTflex^®^ rapid directional qRNA-Seq kit (PerkinElmer, Waltham, MA, USA) was used for library preparation from the rRNA-depleted sample. All reagents and enzymes were supplied by the kit, and the kit’s manual was followed for the library preparation. RNAs were fragmented enzymatically; then, the first and second cDNA strands were generated. The sample was cleaned using AMPure XP beads; then, it was adenylated using the adenylation mix from the kit. Molecular index adapters were ligated to the cDNA; then, it was washed with XP Beads (according to the manual recommendation). Finally, the sample was amplified with PCR and washed with XP beads. The Illumina MiSeq reagent kit v3 (150-cycle format) was used for the sequencing; 12 pM from the library was loaded onto a flow cell.

### 2.5. Determination of the Quantity and Quality of RNA Samples and Sequencing Ready Libraries

The amount of the purified RNA samples was measured using a Qubit 4 fluorometer (Thermo Fisher Scientific) and Qubit RNA (broad-range (BR), quantitation of total RNAs) or Qubit RNA HS assay kit (polyA (+) and rRNA-depleted RNAs). Agilent TapeStation 4150 was used to determine the quality of the RNA samples. The amount of the sequencing ready libraries and the quality assessment of the Illumina library was evaluated using the Qubit 4 with Qubit dsDNA high-sensitivity (HS) and the TapeStation, respectively.

### 2.6. Data Processing and Analysis

#### 2.6.1. ONT Sequencing

Base-calling of the raw data from MinION sequencing was carried out with the Guppy software v3.4.5 (ONT). The minimap2 software [35] was used with the following options: -ax splice -Y -C5–cs to mapping the raw reads to the ASFV genome (accession number: MN715134.1).

For transcript annotations, mapped reads were analyzed using the LoRTIA software suite v.0.9.9 (https://github.com/zsolt-balazs/LoRTIA, accessed on 20 August 2019), using the following steps: (a) Elimination of sequencing reads resulting from partial RT or PCR, as well as artifacts generated by false priming. Non-trimmed read ends were searched for sequencing adapters for the transcriptional start sites (TSS) or the presence of a homopolymer A sequence for the transcriptional end sites (TESs). The first nucleotide not aligning with the adapter was designated as possible TSS, whereas the last nucleotide not aligning with the homopolymer A was designated as putative TES. Any other read start and end positions were discarded. (b) Putative TSSs and TESs were tested against the Poisson distribution to eliminate random start and end positions caused by RNA degradation. The significance is corrected with the Bonferroni method. Features failing to pass qualifying as local maxima or being present in less than 2 reads or in less than 1‰ of the coverage were eliminated from further analysis. The LoRTIA toolkit accepts SAM or BAM files as input and produces GFF files and other processed files as outputs.

The LoRTIA suit was set as follows:

- For dRNA sequencing and dcDNA sequencing reads: −5 TGCCATTAGGCCGGG --five_score 16 -- check_in_soft 15 −3 AAAAAAAAAAAAAAA --three_score 16 s Poisson–f true.
- For o(dT)-primed cDNA reads: −5 GCTGATATTGCTGGG -- five_score 16 --check_in_soft 15 −3 AAAAAAAAAAAAAAA --three_score 16 s Poisson–f true.

Bam files were visualized using Geneious and IGV tools. The TSSs obtained by Cage-Seq and TESs obtained by p(A)-Seq [12] was mapped to the ASFV_HU_2018 genome using Geneious software.

#### 2.6.2. Illumina Sequencing

The raw Illumina reads were trimmed with the Cutadapt tool [36]. The above-mentioned ASFV reference genome was indexed using STAR aligner v2.7.3a [37] using the following settings: --genomeSAindexNbases 8, followed by the mapping of the reads with default options. The RPKM and TPM values of individual ORFs were determined using Geneious software (RPKM: reads per kilobase pair per million mapped reads; TPM: transcript per kilobase million).

## 3. Results

### 3.1. Comparing the Genomes of Strains ASFV_HU_2018 and Ba71V of ASFV

The genome size of the various ASFV strains can significantly vary [38]. We used ASFV_HU_2018 isolate [22], a very close relative of the highly virulent strain Georgia 2007/1 [39] and compared it to the attenuated Vero cell adapted strain Ba71V used for the most detailed ASFV transcriptome analysis available so far [12]. The homologous regions of the two viruses show ~98% similarity. However, the Ba71V genome lacks 22 genes (mostly MGF members) in 15,143 bps lengths, which is present in our virulent ASFV_HU_2018 strain (Table 1). Transcripts identified from these genes are labeled by an asterisk in Table S1.

### 3.2. Analysis of the ASFP Transcriptome with a Dual Sequencing Approach

In this work, we report the application of the combined use of SRS (Illumina MiSeq) and LRS (ONT MinION) techniques for the analysis of the ASFV transcriptome (Tables S1A,B and S2). We used anchored oligo(dT) primers in the LRS and random oligonucleotides in the SRS approach for priming the RT of the first cDNA strand. Both primers were linked to short adapter sequences in those samples, which were amplified by PCR after the RT. We applied three ONT sequencing methods: cDNA sequencing with or without PCR amplification (the latter is termed dcDNA sequencing) and amplification-free native dRNA sequencing. The Illumina SRS yielded altogether 69,068 reads mapped to the ASFV genome with an average genome coverage of 50.13. Illumina sequencing was primarily used for the validation of novel transcripts and for increasing sequencing precision. The amplified cDNA-Seq produced 126,763 ASFV reads, with an average read length of 458 bps and genome coverage of 304.5. Direct cDNA sequencing generated 8587 viral reads with an average read length of 568 base pairs. Native RNA sequencing yielded 4361 viral reads, with an average read length of 637 bp and with an average genome coverage of 14.6.

**Figure 2:**
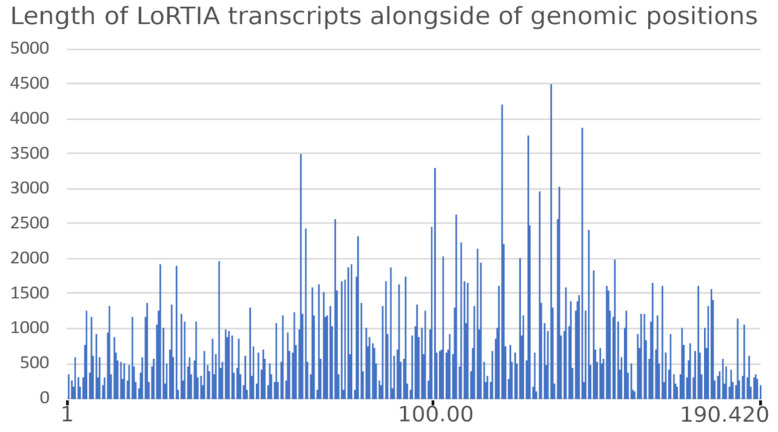
Read-length distribution of LoRTIA transcripts along the ASFV genome.

The average read-length filtered by the LoRTIA software suite was 900.45 bp (read-length alongside the ASFV genome is depicted in Figure 2).

The dataset was deposited and made publicly available earlier [40]. The technical artifacts generated by RT and PCR [41] were removed from further analysis with the help of the LoRTIA tool, albeit in many cases, we were unsure about the nonspecificity of the discarded reads. We used stringent criteria for further filtering the potential spurious transcripts: only those reads were accepted as transcripts, which were detected in at least two independent experiments with precise transcript termini. We applied even more stringent criteria for accepting ncRNAs and truncated RNAs: besides two independent samples, either dcDNA and/or dRNA technique also had to confirm these transcripts. In certain genomic regions, very low coverage was obtained; therefore, the stringent criteria were not applied for the annotation of TSSs and TESs.

**Figure 3:**
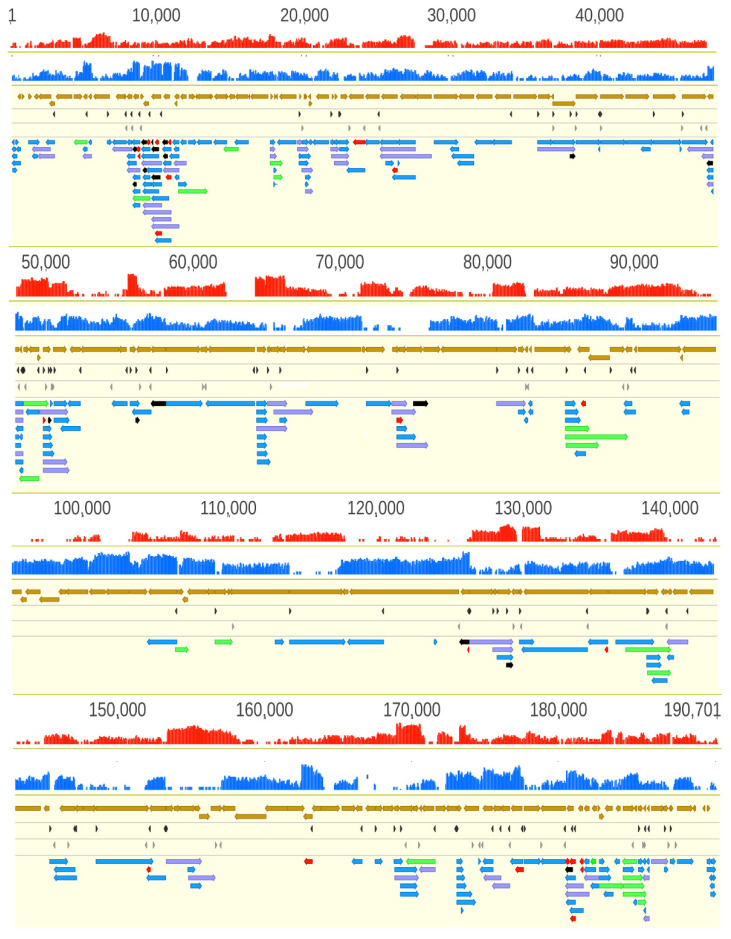
African swine fever virus transcriptome. Panel A illustrates the total transcriptome of ASFV; Panel B. Only those LoRTIA transcripts are depicted of which ratio exceed 2.5% compared to transcripts controlled by the same promoters. The red and the blue histograms represent the coverage of cDNA reads on the two DNA strands. The mRNAs have higher coverage than the antisense RNAs. In principle, asRNAs can be produced from their own promoters, or are the read-through products of convergent genes (→←), or are the consequences of transcriptional start sites (TSS) overlaps of divergently oriented gene products (←→). The proportion of polycistronic transcripts relative to the monocistronic RNAs controlled by the same promoters are shown in Table S7. Color code: light brown arrow-rectangles: coding sequences; dark gray arrow-rectangles: previously annotated TSS; light gray arrow-rectangles: previously annotated TES; black arrow-rectangles: 5′-truncated in-frame ORFs containing transcripts; purple arrow-rectangles: novel polycistronic transcripts; red arrow-rectangles: novel non-coding transcripts; green arrow-rectangles: novel complex transcripts.

We detected a total of 132 LoRTIA and 70 non-LoRTIA TSSs, as well as 137 LoRTIA and 83 non-LoRTIA TESs (Table S3A–D), of which only 98 TSS and 57 TES were annotated earlier [12] In sum, we detected and annotated 311 ASFV RNA molecules (Figure 3, Table S1), including 273 full-length transcripts with precisely identified termini and 38 RNA molecules without accurately annotated TSSs. We obtained 69.01 bp length for an average 5′-untranslated region (UTR) length and 369.31 bp length for the average 3′-UTR lengths.

We were not able to provide precise kinetic statistics on the ASFV transcripts because of the high variance in the data of the parallel experiments that was most probably due to the low and varying infection efficiency of PAM cells by the virus and the relatively low data coverage. However, we could use this dataset to establish whether a certain transcript was expressed at a given time point or not (Table S4).

In Table S5, we compare the transcripts detected by our study using a combined SRS and LRS approach with those detected by Jaing and colleagues using an SRS technique [26]. The sensitivity of their method has remained far behind ours. Since SRS alone is unsuitable to match the corresponding TES and TSS sites of transcripts, they were not able to detect the existence of TSS and TES isoforms and polycistronic transcripts.

### 3.3. Novel Putative Protein-Coding Genes

**Figure 4:**
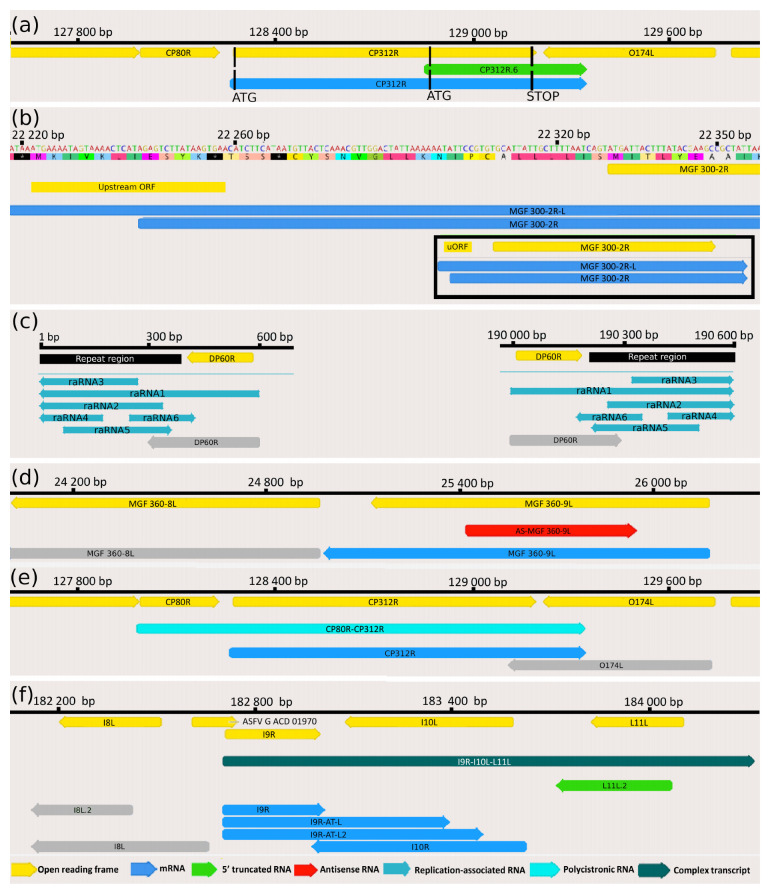
Examples for the ASFV transcript classes (**a**) 5′-truncated mRNAs. This part of the figure illustrates the putative nested mRNAs (CP312R.6; green arrow, embedded into the larger CP312R transcript; blue arrow) containing short 5′-truncated in-frame ORFs within the canonical ORFs (CP312R gene; yellow arrow). (**b**) Transcripts containing upstream ORFs MGF 300-2R transcript and its longer 5′-UTR isoform (MGF 300-2R-L) containing an uORF (yellow rectangle) is illustrated in this picture. The bracketed picture contains two full-length transcript isoforms (blue arrows), as well as the ORF of the gene encoding them and the uORF (yellow arrows). (**c**) Replication origin-associated RNAs This figure illustrates the repeat region at the genomic termini (black rectangle) and the raRNAs (turquoise arrows), which overlap or situated at the vicinity of the Oris, of which the precise location is unknown. (**d**) Antisense RNAs The red arrow indicates an asRNA (AS-MGF 360-9L) overlapping the MGF 360-9L gene (blue arrow) in opposite polarity. (**e**) Polycistronic RNAs CP80R-CP312R bicistronic transcript (light green arrow) comprising two tandemly oriented genes, of which the CP312R is also expressed as a monocistronic RNA molecule (blue arrow). (**f**) Complex transcripts The I9R-I10L-L11L complex transcript (dark green) comprises three genes, of which I9R (encoding three TSS isoforms; blue arrows) stands in an opposite orientation compared to the other two genes (L11L.2 and I10L).

5′-truncated transcripts with in-frame ORFs encoded by putative nested genes. In addition to the transcription of already annotated ORFs, our study detected 16 novel mRNAs coding in-frame short ORFs that are embedded into larger canonical ORFs (having distinct ATGs, but sharing STOP codons) (Table 2). Three additional truncated mRNAs (A137R, CP312R, MGF 100-1L) have also been detected by Cackett and coworkers using a cap analysis of gene expression (CAGE) [12]. We compared our data with the data obtained in this analysis (Table S3A,C). An example for this transcript species is illustrated in Figure 4a.

*Novel intergenic transcripts with small ORFs.* We detected three LoRTIA and four non-LoRTIA small RNA molecules containing short (9–192 bp) ORFs encoded in various intergenic regions (Table S1A,B). Cackett and colleagues have also identified one of these transcripts (pNG6) [12].

### 3.4. Upstream ORF-Containing mRNAs

Recent advances in gene expression studies have shown that more than half of the RNA molecules contain translationally active upstream ORFs (uORFs) located in an upstream position relative to the canonical ORFs [42]. Our study identified 30 novel uORFs in the detected mRNAs, 6 of them overlapped with the main ORFs (Table S2A, Figure 4b) and another 6 of uORFs were only present in the long TSS isoforms of transcripts, but not in the short one. The average distance between the stop codon of uORFs and the ATG of canonical ORFs is 45.76 bps (Table S2A).

### 3.5. Putative Non-Coding Transcripts

Non-coding RNAs (ncRNAs) are specified by RNA genes that are located either at intergenic regions or within protein-coding genes, and they can be encoded by both the positive and negative DNA strands of protein-coding genes. We detected 3 short ncRNAs (sncRNAs) and 42 long ncRNAs (lncRNAs > 200 bp in length), including inter- and intragenic transcripts, antisense RNA (asRNAs), and replication origin-associated RNAs (raRNAs) (Table S1A,B).

This study identified three putative sncRNAs, which were all located at the *intergenic genomic regions* (IG3-MGF 505-9R-MGF 505-10R, IG6-I73R-I329L, and IG7-I329L-I215L) (Table S1B).

The *3′-truncated transcripts* are controlled by the same promoters as the overlapping mRNAs, but these RNA molecules are terminated at sequences preceding the canonical stop codons; therefore, they either do not contain ORFs or code very short ones (Table 3). In ASFV, we detected 22 such transcripts. LoRTIA tool was used for screening of false priming on the A-rich regions of the transcripts and to ensure high reliability.

*Replication origin-associated RNAs* overlap or are located in the vicinity of the replication origins (Oris). These transcripts have been identified in every examined virus [43]; however, their precise functions have not yet been characterized. In herpesviruses, the raRNAs can be non-coding or the longer TSS or TES variants of mRNAs [44]. In ASFV, we detected 6 low-abundance raRNA molecules (Table S1B) at late infection times (12 to 20 h), which are encoded at the terminal repeat region (Figure 4c) that are supposedly include the replication origins of this virus. The lack of raRNAs at the early time points of infection may be due to the low general coverage of the viral reads at these stages of the viral life cycle. Two raRNAs are oppositely oriented than the other four transcripts, of which they overlap. The raRNA1 is a TES isoform of the DP60R transcripts, i.e., in contrast to the others, which are non-coding, it is an mRNA. However, due to the low abundance of these non-LoRTIA transcripts and the low average coverage, we cannot exclude that the TSSs of raRNA2-4 are the same as those of DP60R and raRNA1. Likewise, for the same reason, it is also possible that raRNA5 and six shares a TSS, but since they have different TESs, they are obviously distinct RNA molecules.

*Antisense RNAs* can, in principle, be controlled by distinct promoters, or they can be generated by transcriptional read-through from adjacent or distal convergent genes or by the overlap of divergently oriented genes. In the latter two cases, antisense sequences only make up a part of an mRNA. Our LRS approach detected seven asRNA fragments with identified TESs, but unannotated TSSs (Figure 4d). However, our SRS approach detected a pervasive asRNA expression along the entire ASFV genome. It is assumed that the majority of these transcripts are generated by occasional transcriptional read-throughs from the neighboring convergent genes or by the overlaps of the 5′-UTR of the divergently oriented transcripts, but asRNA may also be controlled by their own distinct cis-regulatory sequences, such as in Figure 4d.

### 3.6. Transcript Isoforms

*TSS isoforms* (variants) contain the same ORFs but differ in the length of their 5′-UTRs and are usually controlled by distinct promoters. We obtained a comparatively low diversity of TSSs, for which the reason may be the relatively low data coverage and the stringent criteria used for the identification of novel transcripts (Table S6). We detected 16 TSS isoforms using the LoRTIA tool (Table S1A, note: #), of which 14 were longer and 2 shorter than the canonical transcripts. We could only confirm one (MGF 300-2R) out of the 8 TSS isoforms reported by Cackett and colleagues [12], but we detected two additional TSS isoforms in their CAGE-seq non-primary TSS list (D345L, C147L).

*TES isoforms* contain the same TSS and ORFs but have distinct poly(A) signals and 3′-end transcript terminals. In this work, we identified 220 TESs (Table S3B,D) and 57 TES (Table S1A, note: ▪) isoforms. Only a few of the TES isoforms (ASFV G ACD 00,600-A224L-AT-L, A151R-AT-S2, I73R-AT-L4, I215L-AT-S, MGF 360-18R-DP71L-DP96R-AT-L) were detected by Cackett and colleagues [12] non-primary p(A)-Seq data. This study did not identify any *spliced transcripts* of ASFV.

### 3.7. Polycistronic Transcripts

Until now, it has been widely assumed that the ASFV genome encodes almost exclusively monocistronic transcripts [9,10,12]. Here we show that it is not the case since our study revealed extensive polycistronism in the ASFV transcriptome. Altogether, 41 bicistronic, 8 tricistronic, and 2 tetracistronic transcripts have been detected using ONT sequencing techniques (Figure 4e) (Table 4). Many of the upstream genes of these multigenic transcripts can also be expressed individually, such as MGF 110-7L-MGF 110-5L-6L-MGF 110-4L, I7L-MGF 100-3L-MGF 100-1L).

### 3.8. Complex Transcripts

Our LRS study demonstrated that ASFV expresses complex transcripts, which, per definitionem, contain at least one gene with opposed polarity in a polygenic transcript (e.g., →←→). We identified a total of 22 complex transcripts (Figure 4f, Table 5).

### 3.9. Transcriptional Overlaps

**Figure 5:**
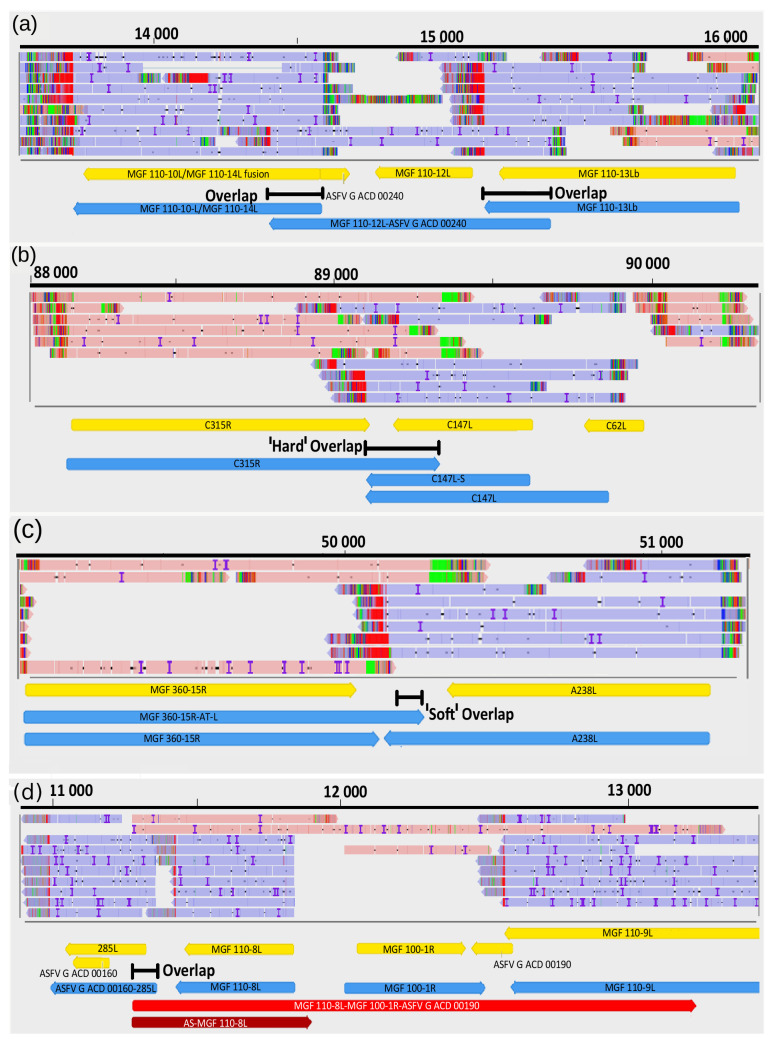
Examples for transcription overlaps of ASFV RNA molecules. (**a**) parallel overlaps, (**b**) convergent "hard" overlap (**c**) convergent "soft" overlap, (**d**) divergent overlap.

We detected 540 parallel, 60 convergent and 19 divergent overlaps (Figure 5). The average lengths of overlaps are as follows: head-to-tail overlaps, 595.3 bp; tail-to-tail overlaps, 319.4 bp; and head-to-head overlaps, ~325.1. The uncertainty of the size of divergent overlaps comes from the fact that most of them were detected by dRNA-Seq, which produces sequencing reads inherently missing 15−35 bp from the 5′-UTRs of the transcripts (Table S2B). Transcriptional overlaps can be formed by transcriptional read-throughs between adjacent genes that can stand in tandem or in convergent orientation relative to each other. In many cases, tandem gene clusters encode 3′ co-terminal transcripts. The upstream genes in these clusters may also have their own transcription termination; that is, they also can produce monocistronic transcripts. Production of mono- or multigenic transcripts from the same gene is controlled by the transcription termination sequences. The frequency of transcriptional read-throughs is shown in Table S7A,B. The ratio of a bicistronic transcript to a monocistronic RNA controlled by the same promoter is corresponds to the efficiency of transcriptional read-through.

## 4. Discussion

African swine fever virus is one of the most important animal pathogens causing severe losses in animal husbandry. Despite its economic significance, no effective prevention therapy (e.g., vaccine) against this virus is available yet. Understanding the architecture of viral transcriptome is not solely an academic issue because drug or vaccine development is essentially dependent on our knowledge of the fundamental molecular mechanisms.

Here we report the first long-read sequencing study on the ASFV transcriptome, which also applies a short-read sequencing approach.

The RT and the PCR may generate technical artifacts, such as nonspecific binding of oligo(dT) or PCR primers and template switching. In addition to poly(A) tails, oligo(dT) primers may also hybridize to A-rich regions of RNA molecules and thereby produce spurious transcripts. Additionally, it has been shown that cDNA library preparation of ONT protocol induces read truncation for transcripts containing internal runs of T’s [41]. However, the combination of various techniques, including LRS and SRS platforms, results in practically error-free, high-coverage, full-length transcription reads, and in our case, this approach yielded the most detailed wild-type ASFV transcription map in natural target cells so far. This analysis revealed numerous novel genes, transcripts, and transcript isoforms (Tables S1 and S2) and ascertained the transcription start and end positions for most of the ASFV transcripts (Table S3). Additionally, in contrast to earlier views that ASFV expresses mainly monocistronic transcripts [9,10,12], our work demonstrates the widespread expression of multigenic RNA molecules (including polycistronic and complex transcripts, along the entire viral genome (Table 4). This important finding should initiate additional research to clarify the role of these transcripts in the transcriptional and translational regulation of the ASFV genome.

The differences between the amounts of transcripts (Table S4) at the same time points in the infected cells of the two animals are somewhat surprising. These variances might originate from statistical uncertainty arising from low data coverage stemming from low infection efficiency. Alternatively, they can represent real differences in macrophages originated from different genetic and immunological backgrounds. Some recent observation may support the latter case: single-cell mRNA sequencing of tissue-resident leukocytes have revealed unexpectedly high levels of heterogeneity among macrophages [45]. At the same time, different subsets of macrophages (moMΦ, moM1 and moM2) show different sensitivity toward ASFV and react with altered cytokine and surface marker production [46]. Hence, it is also possible that the plasticity of the viral transcriptome observed in the parallel experiments is the resultant of the altered interactions of the viral transcription machinery with the discrete sets of macrophages.

Our investigations revealed that the organization of the ASFV transcriptome is more similar to those of the poxviruses than it was expected [47]. Additionally, we demonstrated that, due to a large number of co-terminal transcripts, the ASFV transcriptome also resembles a certain extent those of herpesviruses [17] and baculoviruses [15]. The herpesvirus tandem genes tend to express transcripts with co-terminal poly(A) in the following pattern: abcd, bcd, cd and d, where “a” is the most upstream and “d” is the most downstream gene within the tandem gene cluster. Our study revealed a similar organization of ASFV transcripts; however, most of the ASFV genes also produce monocistronic transcripts (Table 4). Polycistronic transcription is widespread in prokaryotic organisms and in certain viruses but is rare in eukaryotes. In bacteria and bacteriophages, the Shine–Dalgarno sequences control the translation of downstream genes on multigenic transcripts, whereas some small genome viruses developed a variety of mechanisms to tackle this problem, which include the use of internal ribosome entry site (IRES) sequences, ribosomal frameshifting, or leaky ribosomal scanning [48]. However, the function of polycistronism in large DNA viruses is not clearly understood because the translation initiation in these organisms is cap-dependent; therefore, the downstream genes on multigenic transcripts are most probably untranslated. Only a few exceptions to this rule have been discovered so far, which include the translation of uORFs in addition to the downstream canonical ORFs [49,50]. The function of these transcripts is currently unknown. They may play a regulatory role in transcription and/or translation, or they are mere by-products of a transcription interference mechanism [16]. It cannot be excluded that they represent transcription noise without any function. Many of these transcripts are produced in low abundance, which might indicate this latter scenario. Future investigations have to clarify this issue. We note here that ONT sequencing significantly underestimate the proportion of polycistronic transcripts relative to the monocistronic RNAs due to the size-preference of this approach. A larger data coverage would probably increase the types of these transcripts, which is indicated by the fact that 13 transcripts were only identified by dRNA-Seq that produced the longest average read lengths. 

We identified a number of uORFs (Table S2A) and found that none of them are expressed on separate RNAs independently of the canonical ORFs. Although the importance of uORFs is not yet very well understood, it has been suggested that they may have an important role in the regulation of gene expression [51,52,53]. Additionally, we demonstrated a potentially intriguing variance in a few cases, namely, that only the longer TSS isoforms of transcripts contain uORFs, but not the shorter ones. We observed a similar phenomenon in human cytomegalovirus [16]. Potentially, the two transcript isoforms may provide a distinct translational regulation of the same gene at different stages of viral infection.

It has recently been shown in several viruses that transcripts embedded into a larger host gene and containing 5′-truncated in-frame ORFs within larger canonical ORFs –, of which they share stop codons—are much more common than it has previously been thought [15,16,54]. The TSSs of some of these transcripts were also present in Cackett and colleagues’ non-primary TSS list [12]. Using LRS, 19 novel short in-frame ORFs were also identified with the potential to code the amino terminus truncated versions of canonical proteins (e.g., A151R full-length transcript and A151R.3 5′ truncated transcript) (Table S1). The SRS approach is inefficient in the discovery of the nested genes; that is why they had gone undetected before (Figure 3, Table S3). Further experiments are needed to demonstrate whether the coding potential of these putative nested genes is realized in translation in every annotated transcript. Another class of putative protein-coding genes that were detected in intergenic locations specifies relatively short transcripts containing small ORFs. Cackett and colleagues have also identified one of these transcripts (pNG6). Further investigations must be carried out to ascertain whether these small transcripts are translated.

We also detected various classes of non-coding transcripts, including intra- and intergenic transcripts and antisense RNAs. The asRNAs are transcriptional read-through products of convergent genes or are generated by divergent overlaps, or they can have their own promoters as in Figure 4d. We also identified six low-abundance replication origin-associated RNA (raRNA) molecules (Table S1B). A recent study has demonstrated that 72% of mammalian raRNAs are associated with active promoters, which controls the expression of protein-coding or non-coding genes [55]. These transcripts have also been detected in viruses [43]. It has been demonstrated in human BK polyomavirus that a raRNA, by binding simultaneously to both sense and antisense DNA strands within the Ori region, significantly inhibits the replication of the virus through interfering with the RNA primer synthesis [56]. Further analyses are needed to ascertain the precise function of these transcripts in ASFV and other viruses. The fact that these ASFV transcripts are polyadenylated suggests that they are likely important as RNA molecules that are able to exert their effect in small copy numbers.

Transcripts can form co-oriented, convergent and divergent overlaps. Polycistronic ASFV transcripts represent a parallel (co-oriented) overlap of RNA molecules encoded by tandem genes. The distinctive feature of ASFV convergent overlaps compared to herpesviruses is the large number of “hard” overlaps (all transcripts overlap with the convergent transcripts for a gene pair) and the large proportion of overlaps in the “soft” overlaps (only a certain ratio of transcripts forms overlaps as a result of transcriptional read-through) (Figure 5). Another difference between the ASFV and herpesvirus transcripts is that the ASFV genes within a tandem gene cluster can produce both mono- and polycistronic RNA molecules, whereas most of the herpesvirus transcripts share common TESs. This genomic organization suggests an important role of transcriptional interference in the regulation of genome-wide gene expression of ASFV [57].

Besides the confirmation of already annotated transcripts [12], our study identified several novel TSSs, TESs (110 TSSs, 163 TESs), and RNA molecules. No spliced ASFV transcripts were detected in this work.

In sum, our work provides a near-complete assembly of the ASFV transcriptomic atlas. Understanding the genetic regulation provides new insights into the virus pathogenicity. Our results in ASF research imply that targeting the expression of raRNAs may provide an effective antiviral strategy. Another importance of our results is the discovery that the putative nested genes represent an unexpectedly large fraction of coding sequences, which may also be the case in other viruses. Furthermore, this work highlights the necessity for the use of a multiplatform approach in transcriptomic studies.

## Figures and Tables

**Table 1:** List of genes lacking from the Ba71V genome. This table summarizes the lacking ORFs’ genomic coordinates.

ORF	ATG (+)/Stop (−)	ATG (−)/Stop (+)	Strand
DP60R	403	582	−
285L	11,042	11,326	−
MGF 110-8L	11,455	11,838	−
MGF-100-1R	12,056	12,430	+
MGF 110-9L	12,589	13,461	−
MGF 110-10L/MGF 110-14L	13,752	14,570	−
MGF 110-12L	14,760	15,119	−
MGF 110-13Lb	15,205	16,029	−
MGF 360-4L	16,210	17,373	−
MGF 360-6L	18,188	19,315	−
MGF 360-10L	26,367	27,404	−
MGF 360-11L	27,432	28,493	−
MGF 505-1R	28,701	30,296	+
MGF 360-12L	30,349	31,401	−
MGF 360-13L	31,562	32,623	−
MGF 360-14L	32,808	33,881	−
I7L	181,681	181,989	−
I8L	182,203	182,514	−
I9R	182,709	182,999	+
I10L	183,075	183,587	−
I11L	183,826	184,107	−
DP60R	190,010	190,189	+

**Table 2:** List of the 5′ truncated transcripts.

Name	TSS (+)/TES (−)	TSS (−)/TES (+)	Strand
MGF 110-3L.3-AT-L	8183	8458	−
MGF 110-3L.3	8210	8458	−
MGF 110-4L.3-AT-L	8785	9146	−
MGF 110-4L.3	8902	9146	−
MGF 110-5L-6L.2-AT-L2	9468	9961	−
MGF 110-5L-6L.1	9468	10,064	−
MGF 110-5L-6L.6	9468	9585	−
MGF 110-7L.1-AT-L	10,220	10,563	−
MGF 110-7L.1	10,270	10,563	−
MGF 505-4R.13	37,956	38,295	+
A224L.5	47,236	47,621	−
A151R.3	49,961	50,173	+
A137R.4	55,895	56,146	+
F334L.2	56,946	57,937	−
EP402R.2	74,801	75,785	+
CP204L.2	125,685	126,344	−
CP312R.6	128,878	129,347	+
MGF 100-1L.2	180,386	180,891	−
I8L.2	182,120	182,425	−

**Table 3:** List of the non-coding transcripts.

Name	TSS (+)/TES (−)	TSS (−)/TES (+)	Strand
nc-MGF 110-3L	8455	8680	−
nc-MGF 110-3L-AT-S	8526	8680	−
nc-MGF 110-4L	9146	9368	−
nc-MGF 110-5L-6L	9705	9961	−
nc-MGF 110-5L-6L-L	9705	10,162	−
nc-MGF 110-7L-AT-L	10,481	10,814	−
nc-MGF 110-7L	10,619	10,814	−
nc-MGF 300-4L	23,203	23,976	−
nc-MGF 360-9L	25,832	26,183	−
nc-A151R	49,646	49,784	+
nc-EP152R-EP153R	73,637	74,032	+
nc-c275L	86,209	86,487	−
nc-CP204L	126,247	126,344	−
nc-NP419L	135,550	135,755	−
nc-H359L	151,958	152,195	−
nc-E184L	162,670	163,182	−
nc-DP238L	176,989	177,547	−
nc-MGF 100-1L	180,386	180,613	−
nc-MGF 100-3L-MGF 100-1L-AT-L2	180,671	181,084	−
nc-MGF 100-3L-MGF 100-1L-AT-L3	180,743	181,084	−
nc-MGF 100-3L	181,362	181,585	−
nc-MGF 100-3L-AT-S	181,408	181,585	−

**Table 4:** List of polycistronic transcripts.

Name	TSS (+)/TES (−)	TSS (−)/TES (+)	Strand	RNA Type
MGF 360-1La-KP93L	2625	1367	−	Bicistronic
MGF 360-1La-MGF 360-1Lb	2914	1752	−	Bicistronic
L60L-L83L	5405	4819	−	Bicistronic
MGF 110-3L-MGF 110-2L	8680	7800	−	Bicistronic
MGF 110-5L-6L-MGF 110-4L	10,162	8785	−	Bicistronic
MGF 110-5L-6L-MGF 110-4L-AT-S	10,162	8902	−	Bicistronic
MGF 110-7L-MGF 110-5L-6L	10,814	9468	−	Bicistronic
285L-MGF 110-7L	11,372	10,270	−	Bicistronic
MGF 110-8L-ASFV G ACD 00160-285L	11,854	10,991	−	Bicistronic
ASFV G ACD 00290-ASFV G ACD 00350-AT-L	17,530	17,963	+	Bicistronic
QGV56997.1-ASFV G ACD 00320	19,383	19,699	+	Bicistronic
ASFV G ACD 00320-ASFV G ACD 00330	19,482	20,130	+	Bicistronic
ASFV G ACD 00330-X69R	19,712	20,421	+	Bicistronic
ASFV G ACD 00350-X69R	19,930	20,421	+	Bicistronic
ASFV_G_ACD_00290-MGF 300.5-2R	21,682	22,200	+	Bicistronic
MGF 300.5-2R-MGF 300-2R	21,935	22,888	+	Bicistronic
MGF 360-10L-MGF 360-9L	27,409	24,983	−	Bicistronic
MGF 505-3R-MGF 505-4R	35,730	38,296	+	Bicistronic
ASFV G ACD 00600-A224L-AT-L2	48,230	45,900	−	Bicistronic
ASFV G ACD 00600-A224L-AT-L	48,240	46,868	−	Bicistronic
ASFV G ACD 00600-A224L	48,240	47,236	−	Bicistronic
A151R-MGF 360-15R-L	49,395	51,279	+	Bicistronic
A151R-MGF 360-15R	49,646	51,269	+	Bicistronic
A151R-MGF 360-15R-AT-L	49,646	51,387	+	Bicistronic
EP152R-EP153R	73,293	74,365	+	Bicistronic
EP152R-EP153R-AT-L	73,293	74,953	+	Bicistronic
EP153R-EP402R	73,637	75,785	+	Bicistronic
M448R-C129R	80,455	82,407	+	Bicistronic
CP530R-CP80R	126,388	129,348	+	Bicistronic
CP80R-CP312R	127,979	129,347	+	Bicistronic
D339L-DP79L	141,202	139,812	−	Bicistronic
H108R-H233R	154,764	156,604	+	Bicistronic
EP296R-E111R-L	168,803	170,408	+	Bicistronic
I267L-E66L	171,549	170,453	−	Bicistronic
I177L-I215L	176,031	174,820	−	Bicistronic
I196L-I177L	176,620	175,414	−	Bicistronic
MGF 100-3L-MGF 100-1L	181,585	180,386	−	Bicistronic
I8L-I7L	182,657	181,655	−	Bicistronic
ASFV G ACD 01940-ASFV G ACD.5 01940	186,102	185,635	−	Bicistronic
ASFV G ACD 01940-ASFV G ACD.5 01940-AT-L	186,102	185,686	−	Bicistronic
MGF 360-19Ra-MGF 360-19Rb	186,225	187,363	+	Bicistronic
MGF 110-2L-ASFV G ACD 00090-MGF 110-1L	8153	6830	−	Tricistronic
MGF 110-7L-MGF 110-5L-6L-MGF 110-4L	10,814	8902	−	Tricistronic
285L-MGF 110-7L-MGF 110-5L-6L	11,372	9468	−	Tricistronic
QGV56997.1-ASFV G ACD 00320-ASFV G ACD 00330	19,383	20,130	+	Tricistronic
ASFV G ACD 00290-MGF 300.5-2R-MGF 300-2R	21,697	22,890	+	Tricistronic
MGF 360-11L-MGF 360-10L-MGF 360-9L	28,482	24,983	−	Tricistronic
K78R-K196R-K145R	64,869	66,167	+	Tricistronic
I7L-MGF 100-3L-MGF 100-1L	181,993	180,386	−	Tricistronic
K205R-K78R-K196R-K145R	64,142	66,167	+	Tetracistronic
H171R-H124R-H339R-H108R	153,246	155,658	+	Tetracistronic

**Table 5:** List of the complex transcript.

Name	TSS (+)/TES (−)	TSS (−)/TES (+)	Strand
L83L-KP177R	4203	5129	−
MGF 110-4L-MGF 110-3L	8210	9368	−
MGF 110-8L-MGF 100-1R-ASFV G ACD 00190	11,282	13,246	+
MGF 110-12L-ASFV G ACD 00240	14,412	15,385	−
ASFV G ACD 00290-ASFV G ACD 00350-ASFV G ACD 00300	17,530	18,383	+
ASFV G ACD 00350-ASFV G ACD 00300	17,782	18,383	+
A240L-A104R	48,012	49,331	−
A104R-A240L-A118R	48,308	49,985	+
C122R-C257L	85,124	86,738	+
C122R-C257L-C475L-C315R	85,124	89,338	+
C122R-C257L-AT-L	85,144	87,363	+
B125R-B117L	106,380	107,270	+
B263R-B66L	109,076	110,269	+
D205R-D129L-L	137,005	140,038	+
D205R-D129L	138,451	140,032	+
I267L-E66L-E111R	169,555	171,535	−
I9R-I10L-L11L	182,703	184,322	+
DP71L-MGF 360-18R	184,247	185,256	−
MGF 360-18R-DP71L-DP96R	184329	185647	+
MGF 360-18R-DP71L-DP96R-ASFV G ACD.5 01940	184,329	185,895	+
MGF 360-18R-DP71L-DP96R-AT-L	184,329	185,733	+
DP96R-ASFV G ACD.5 01940	185,335	185,895	+

## Data Availability

The sequencing datasets generated during this study are available at the European Nucleotide Archive’s SRA database under the accession PRJEB36723. The LoRTIA software suite is available on GitHub: https://github.com/zsolt-balazs/LoRTIA, accessed on 20 August 2019.

## Supplementary Materials

The following are available online at https://www.mdpi.com/1999-4915/13/4/579/s1, Table S1. List of the ASFV transcripts identified in this study. Table S2. Summary tables. (A) List of upstream (u)ORF-containing mRNAs. This sheet contains information about the exact location of uORFs. (B) Overlapping transcript list. This table shows the lengths and types of overlaps between the overlapping RNAs. (C) Lengths of UTR sequences of the ASFV transcripts. (D) Summary table of the ASFV ORFs. This panel shows detailed information about the ORFs, including their genomic locations and lengths, as well as the Illumina RPKM and TPM values for them. RPKM—reads per kilobase pair per million mapped reads; TPM—transcript per kilobase million; Table S3. The list of the novel TSSs and TESs determined in our study and the comparison of our data with those of Cackett et al. [12] obtained using Cage-Seq and p(A)-Seq. The count of reads validating the given TSS or TES positions are also enlisted; Table S4. ASFV transcripts along with their expression dynamics; Table S5. Comparison of ASFV transcripts identified by short- and long-read sequencing techniques; Table S6. Summary statistics of the sequencing libraries; Table S7. The efficiency of transcriptional read-through between neighboring gene pairs.
