# Supplementary material for: Combined Short and Long-Read Sequencing Reveals a Complex Transcriptomic Architecture of African Swine Fever Virus
Source: Viruses. 2021 Mar 30;13(4):579. doi: 10.3390/v13040579 (PMC8103240; doi:10.3390/v13040579)
Supplement: Supplementary file 1 [file viruses-13-00579-s001.zip › viruses-13-00579-s001/viruses-1130753 supplementary legend.pdf]

Table S1. **List of the ASFV transcripts identified in this study**. The sheets of this table contain detailed information about the annotated transcripts, including their genomic positions. Those transcripts which lack from the BA71V strain, are labeled with asterisk. **A**. Transcripts annotated by the LoRTIA software tool. The 5′ and 3′ UTR transcript isoforms are labeled with # and ▪, respectively in the column 'Note'. **B**. Non-LoRTIA transcripts.

Table S2. **Summary tables**. **A**. List of upstream (u)ORF containing mRNAs. This sheet contains information about the exact location of uORFs. **B**. Overlapping transcript list. This table shows the lengths and types of overlaps between the overlapping RNAs. **C**. Lengths of UTR sequences of the ASFV transcripts. **D**. Summary table of the ASFV ORFs. This panel shows detailed information about the ORFs, including their genomic locations, and lengths, as well as the Illumina RPKM and TPM values for them. RPKM - reads per kilobase pair per million mapped reads; TPM - transcript per kilobase million.

Table S3. **The list of the novel TSSs and TESs determined in our study and the comparison of our data with those of Cackett et al. [12] obtained using Cage-Seq and p(A)-Seq**. The count of reads validating the given TSS or TES positions are also enlisted. **A**. The list of transcription start sites identified by LoRTIA package. Transcripts of which the TSSs were confirmed by CAGE-seq technique (Cackett et al.) are labelled with asterisk. **B**. The list of transcription end sites identified by LoRTIA package. Transcripts of which the TESs were confirmed using polyA-sequencing (Cackett et al.) are labelled with asterisk. **C**. The list of transcription start sites identified manually. Transcripts of which the TSSs were confirmed by CAGE-seq technique (Cackett et al.) are labelled with asterisk. **D**. The list of transcription end sites identified manually. Transcripts of which the TESs were confirmed by polyA-sequencing (Cackett et al.) are labelled with asterisk.

Table S4. **ASFV transcripts along with their expression dynamics.** **A**. The list of transcripts identified by LoRTIA software. Transcripts which were confirmed by dRNA sequencing are labelled with asterisk. **B**. The list of transcripts identified manually.

Table S5. **Comparison of ASFV transcripts identified by short - and long-read sequencing techniques**. The canonical mRNAs are written with bold letters. SRS detected transcriptional activity from the genes but did not identify full-length transcripts, that is why more than 311 transcripts are indicated in this Table.

Table S6. **Summary statistics of the sequencing libraries**

Table S7. **Efficiency of transcriptional read-through between neighboring gene pairs**. Read-through efficiencies can be determined by calculating the proportion of polycistronic and monocistronic transcripts. These are semi-quantitative data because ONT sequencing is biased toward short transcripts, and therefore the amount of long multicistronic RNAs is significantly underestimated. Thirteen transcripts were only detected by dRNA-Seq for which the reason is that this approach generated the longest average read lengths. Likewise, the exact TSS of these 13 RNA molecules could not be precisely annotated.
